# Treatment of sleeping disorders should be considered in clinical management of Parkinson's disease

**DOI:** 10.3389/fnagi.2014.00273

**Published:** 2014-10-09

**Authors:** Altair Brito dos Santos, George E. Barreto, Kristi A. Kohlmeier

**Affiliations:** ^1^Department of Biological Sciences, Universidade Estadual do Sudoeste da BahiaBahia, Brazil; ^2^Departmento de Nutrición y Bioquímica, Facultad de Ciencias, Pontificia Universidad JaverianaBogotá DC, Colombia; ^3^Department of Drug Design and Pharmacology, Faculty of Health and Medical SciencesUniversity of Copenhagen, Denmark

**Keywords:** REM-sleep behavior disorder, mesopontine cholinergic cell group, neurodegeneration, restless leg syndrome, excessive daytime sleepiness

Following two decades of research after the first clinical reports that a sleep disturbance can precede signs and motor symptoms of Parkinson's disease (PD) (Schenck et al., 1996), studies examining sleep at the cellular and systems-wide level in PD-diagnosed and preclinical PD patients have lead to the belief among sleep researchers that disturbed sleep occurs long before PD diagnosis and could be a predictor of the disease [For example, see (Postuma et al., 2009a,b; Hansen et al., 2013)]. More excitingly, these studies have led to the tantalizing conclusion that aggressive treatment of sleeping disorders could actually delay progression of PD. However, while clinicians fully expect presence of sleeping disorders in their PD-diagnosed patients and manage these complaints (Jahan et al., 2009; Diederich and McIntyre, 2012; Klingelhoefer et al., 2014), the possibility of exploitation of the link between PD and sleeping disorders for pre-clinical PD diagnosis or treatment approaches designed to delay motor symptoms of PD has not gone far outside the sleep research field. Despite the fact that there is a significant literature comprised of human epidemiological and post-mortem studies, as well as animal studies, which have correlated varying manifestations of sleep disorders or changes in neural areas controlling sleep, as preceding or simultaneous with PD [For example, see (Arnulf et al., 2002, 2008; Korczyn, 2006; De Cock et al., 2008; Stavitsky and Cronin-Golomb, 2011)], clinicians fail to strongly consider that a patient presenting with a sleeping disorder has a higher risk of later life development of PD and that, therefore, these patients should be carefully monitored for eventual onset of the classic signs of neurodegenerative diseases such as PD. Nor is it standard practice to suggest prophylactic approaches in these patients to curb onset of PD.

Why is the link between sleep disorders and eventual appearance of the classic signs of PD and the possibility that treatment could delay PD onset not been made clinically? Clinical acceptance of this link and of the idea that treatment of sleeping disorders could stave off PD onset would be facilitated by positive findings from longitudinal studies with human patients focused on elucidating whether there exists a strong association and/or causality between sleeping disorders and PD and whether aggressive management of the sleeping disorder appears to delay appearance of classic PD signs. However, performing these studies raises ethical issues, especially if, in an attempt to provide compelling evidence for causality, preventative interventions are given to one group of subjects and not another. Baring these studies, convincing evidence could be gained of the link from improved monitoring of the neurological progress of patients with sleeping disorders in combination with imaging studies focused on full characterization of changes in neural regions controlling sleep which occur in patients eventually presenting with PD.

But, the question is which behavioral state disorder(s) should be monitored for predictive and possibly therapeutic value in PD. Arguably, the most well-established association of presence of a sleeping disorder preceding any defining signs of PD is the occurrence of Rapid Eye Movement (REM)-Sleep Behavior Disorder (RBD), which is a motor disorder characterized by aberrant motor activity during the phase of sleep known as REM, years before PD diagnosis (Schenck et al., 2002; Schenck and Mahowald, 2002; Diederich and McIntyre, 2012). In a group of RBD patients, epidemiological studies revealed that nearly 80% of those eventually developed alpha-synucleinopathies such as PD, leading to the suggestion that RBD may represent a prodromal PD sign, indicating a phase present 2–13 years before appearance of the more classic signs of motor symptomology (Schenck et al., 1996; Tippmann-Peikert et al., 2006; Postuma et al., 2009a,b; Fantini et al., 2011). Animal models of PD have contributed compelling evidence that there is a link between sleep and PD, and have shown motor changes associated with REM reminiscent to those of RBD in humans (Verhave et al., 2011). Since RBD can occur years before the onset of PD motor symptoms, while presence of motor disturbances while awake is currently the principal diagnosis criteria of PD, an even better understanding of the disturbances of the motor control of sleep in RBD preceding motor dysfunction in wakefulness gained from longitudinal studies in humans and studies using animal models of PD, could pave the way toward development of a robust, reliable preclinical sleep-related marker of eventual onset of PD.

However, RBD is not the only sleeping disorder associated with PD, as sleep-related symptomology beyond RDB is reported to be present in 60–90% of PD and although the temporal relationship with PD has not been widely studied in all of these disorders, these symptoms may actually be present quite a bit earlier than appearance of waking state motor symptoms. Restless leg syndrome (RLS), an uncontrollable urge to move the legs in sleep, was found in patients pre-symptomatic of PD and from the first prospective study of its type, severe RLS was found to be associated with a higher risk of later life development of PD (Wong et al., 2014). Excessive daytime sleepiness (EDS), which is characterized by a persistent sleepiness due to a lack of adequate sleep, but can also occur when adequate night-time rest is achieved, has been considered as a premotor marker for the development of PD (Abbott et al., 2005; Gao et al., 2011) and recent findings in animal models suggest that occurrence of EDS could participate in development of the pathophysiology associated with PD (Barraud et al., 2009; Verhave et al., 2011). Sleep is controlled in a circadian fashion, and studies have indicated that there are early changes in the circadian control of sleep associated with PD (Breen et al., 2014), which may precede evidence of motor dysfunction. What all these aforementioned disorders have in common is that when present, they lead to inadequate sleep, which is interesting in light of recent theories in the sleep field regarding the function of sleep as a time of brain “clean up.” These theories are based in large part upon findings in rodents that the glymphatic space, the channel between neurons and glia, widened during sleep (Xie et al., 2013) and that ß-amyloid levels in sleep deprived mice were higher than those in control animals (Kang et al., 2009). Therefore, later life development of symptoms associated with neurodegenerative diseases may source, in part, from presence of sleeping disorders which lead to inadequate rest underlying a reduced opportunity for flushing of degeneration-associated proteins from the extracellular neuron-glial space. When taken together, the data suggest that better studies need to be conducted to determine to what extent these disorders affecting sleep are associated with, and precede, the motor symptoms of PD to determine their usefulness as a clinical diagnostic tool, and whether their management would be fruitful in abatement of PD development.

Based on data presently available, sleep disorders appear to precede the motor deficits of PD by several years; therefore, it is parsimonious to assume that these manifestations of aberrant control of the phenomenology of sleep are directly due to degeneration in neural regions importantly involved in control of sleep. Evidence for degeneration in neural regions that are known to control sleep behavior that occurs prior to loss of dopamine-containing striatal neurons, which is considered the gold-standard neural signature of PD, would lend support to a clinical conclusion that sleeping disorders can be used as diagnostic tools for PD. Such evidence exists. Degeneration has been shown to be present early in PD in a group of state-related cholinergic neurons within the brainstem. Post-mortem studies of PD patients have shown a significant loss of cholinergic neurons of the mesopontine tegmentum (MPCH) which via an extensive cholinergic innervation of rostral and caudal targets is believed to control phenomenology of REM, including motor tone and cortical activity, and data suggests that the degree of loss of the cholinergic neurons of the MPCH could be associated with the severity of PD symptoms (Zweig et al., 1989). Loss of MPCH neurons would be predicted to manifest in motor behavior dysfunctions during REM similar to those seen prior to PD, such as RBD. Interestingly, the MPCH has been the target of therapeutic stimulation to successfully alleviate gait disorders in fully developed PD believed to be due, in part, to loss of coherent oscillatory activity in striatal and cortical regions due to MPCH dysfunction. However, in addition to improving gait, implantation of electrodes for deep brain stimulation of the pedunculopontine tegmentum within the MPCH has also been found to normalize several sleep parameters even up to 1 year post surgery (Peppe et al., 2012). MPCH neurons are not only involved in control of sleep but they are also believed to be importantly involved in generation of gamma activity underlying aroused electrical cortical activity. If MPCH neurons are lost very early in PD, as suggested by the early appearance of abnormal motor behavior in sleep, it also suggests that patients with preclinical signs of PD would show significant aberrations in their ability to direct internal attention and focus on cognitive and motor tasks. However, studies are lacking on early indications of loss of attentional processes in these patients prior to appearance of striatal motor signs.

While it is known from post-mortum studies that degeneration of MPCH neurons, along with other neurons involved in state control, such as arousal-related orexinergic neurons of the hypothalamus (Thannickal et al., 2007), occurs in PD, the etiology and time-line of this cell loss, which likely occurs prior to the hallmark loss of dopamine-containing cells of the striatum is less well-understood. Appreciation of the early appearance of sleep and arousal related symptomology in PD should drive intensive focus in research investigations of disease-related neurodegeneration of the exact course of loss of neurons in regions of the brain controlling state in patients presenting with behavioral state disorders. To this goal, improved imaging technology has the potential to increase our understanding of the relationship of degeneration of the brainstem and the hypothalamus and the appearance of disorders of sleep before emergence of the distinguishing motor signs leading to diagnosis. Hopefully, with continued improvement of this technology, a correlation can be made between non-striatal neuronal degeneration, behavioral state symptomology and PD in an attempt to develop criteria for earlier recognition of a high likelihood of later development of neurodegenerative disease, which could suggest an aggressive, possibly counteracting treatment of the disorder of state.

In light of findings from clinical and animal studies, we are supportive of the opinion that not only is the disorder of RBD an early marker of PD, but that also other behavioral state disorders such as RLS, EDS and circadian system dysfunctions, demonstrate the potential to be early markers of PD and should be exploited as reliable indicators for preclinical PD. Targeted investigations which should include imaging techniques are expected to provide more accurate data upon which clear conclusions of the co-occurrence of sleeping disorders and PD can be drawn, and upon which reasonable underlying mechanisms can be determined. The presence of behavioral state disorders in animal models of PD indicates that more invasive detailed studies of the relationship of dysfunctioning in brainstem nuclei involved in sleep and arousal and the motor control of sleep can be performed, providing more conclusive evidence of the sleep-PD link. When taken together, this work will go a long way in convincing clinicians that sleep disorders are preclinical markers of PD. Such an awareness will likely lead to increased observations by practitioners of the relationship of PD and sleep, which will enhance detection of sleep abnormalities, which will serve to feed-forward and advance the use of presence of these disorders as preclinical markers of PD. Detection of PD before the classic motor signs offers the very real possibility of application of earlier, and perhaps more refined, therapies for both PD and quite possibly, the associated behavioral state disorders appearing before PD motor symptoms. More intriguingly perhaps is the possibility that treatment of disorders of sleep could slow the progression of the disease.

## Conflict of interest statement

The authors declare that the research was conducted in the absence of any commercial or financial relationships that could be construed as a potential conflict of interest.
